# A Novel RFID-Based Sensing Method for Low-Cost Bolt Loosening Monitoring

**DOI:** 10.3390/s16020168

**Published:** 2016-01-28

**Authors:** Jian Wu, Xingmei Cui, Yunpeng Xu

**Affiliations:** 1 School of Mechanical Engineering, Nanjing University of Science and Technology, Nanjing 210094, China; 18551742928@163.com (X.C.); 113101000162@njust.edu.cn (Y.X.); 2 Jiangsu Key Construction Laboratory of Modern Measurement Technology and Intelligent System, Huaiyin Normal University, Jiangsu 223300, China

**Keywords:** guide structure, UHF RFID, bolt loosening

## Abstract

In coal mines, bolt loosening in the cage guide is affected by the harsh environmental factors and cage hoist vibration, leading to significant threats to work safety. It is crucial, to this effect, to successfully detect the status of multipoint bolts of guide structures. This paper proposes a system to monitor bolt status in harsh environments established based on the RFID technique. A proof-of-concept model was demonstrated consisting of a bolt gearing system, passive UHF RFID tags, a reader, and monitoring software. A tinfoil metal film is fixed on the retaining plate and an RFID tag bonded to a large gear, with the bolt to be detected fixed in the center of a smaller gear. The radio-frequency signal cannot be received by the reader if the tag is completely obscured by the tinfoil, and if the bolt is loose, the tag’s antenna is exposed when the gear revolves. A radio-frequency signal that carries corresponding bolt’s information is transmitted by the RFID tag to the RFID reader due to coil coupling, identifying loose bolt location and reporting them in the software. Confirmatory test results revealed that the system indeed successfully detects bolt loosening and comparative test results (based on a reed switch multipoint bolt loosening monitor system) provided valuable information regarding the strengths and weaknesses of the proposed system.

## 1. Introduction

Bolt joints, a common component of large-scale steel equipment and structures, are widely used in aeronautic, civil, mechanical, chemical, and other industries [1,2]. The personal safety of mine workers is directly affected by bolt joints typically applied in coal mine cage guides. Guide structures are responsible for the transportation of several tons of goods from hundreds of meters underground to the ground, and repeated external impact and violent vibration easily cause them to suffer structural fatigue, cracks, and even fracture, followed by reduced tension which loosens the bolts of the cage guide. Because humidity and temperature in a coal mine are typically higher than those on the ground, bolts exposed to the atmosphere are more vulnerable to corrosion by moisture in the air, which can damage the bolts, reduce their tensile force, and loosen them. Besides, improper mounting and a myriad other reasons can likewise result in bolt loosening throughout their service lives. Though bolt joints are mechanically locked, bolt loosening in the guide structure is still effectively impossible to avoid, and even small loosening in the initial stage can grow quite large with time. Therefore, periodic inspection of bolt loosening in cage guides is necessary to protect workers and minimize catastrophic accidents.

At present, bolt loosening is mainly judged by bolt fastening force testing, primarily through the torque wrench method or ultrasonic detection method. The torque wrench method is typically adopted to control bolt fastening force in engineering, specifically, however, it does not readily provide accurate measurement due to friction in the nut and structure. To conduct the ultrasonic detection method, an ultrasonic probe is mounted on the bolt to measure fastening force by ultrasonic echo. However, as the bolt head needs to be smooth to fit an ultrasonic probe, it is difficult to complete the installation properly [3,4,5].

Existing monitoring technology of bolt loosening in cage guides is generally lacking; it is highly time-consuming and expensive because it requires regular manual operation to check and maintain. The harsh environment in the coal mine makes it especially difficult to detect bolt loosening, in addition to the fact that the coal mine cage guide is usually several hundred meters long, making monitoring such a large number of bolts at the same time very difficult. Passive surveillance methods based on RFID techniques have been proposed to solve this problem [6,7,8]. Most of the applications in passive wireless sensing technology are deployed in uncontrollable locations or harsh environments [9,10], and passive monitoring can work well in such situations without hindering the communicating systems. The most important advantage of passive monitoring is that it does not occupy any resources in the original network; wireless communication is triggered spontaneously only when the corresponding node is activated under certain conditions.

In this study, we developed an RFID technique based on passive monitoring [11,12,13] to monitor bolt loosening in cage guides. The remainder of this paper is organized as follows: Section 2 describes the application of RFID to achieve detection of loose bolts, including the RFID detection principle and system components. Section 3 reports our results of testing the signal strength between the RFID tag and reader. Section 4 discusses the software system design. Section 5 focuses on the verification experiments and evaluation of system performance, and tests we ran on a comparable system to monitor multipoint bolt loosening. Finally, the performance of the combined hardware and software is verified in a simulated re-entry environment to determine whether the developed prototype diagnoses bolt loosening with sufficient reliability and accuracy.

## 2. Bolt Monitoring System Hardware Design

### 2.1. UHF Passive RFID Technology

**Figure 1:**
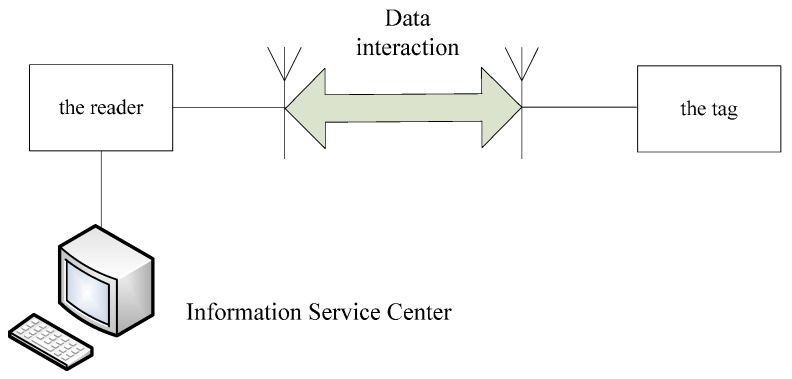
Typical UHF passive RFID system.

Ultra high frequency (UHF) passive radio frequency identification (RFID) technology is an advanced automatic identification technology that shows many advantages, including high recognition distance (3–10 m), recognition accuracy (above 95%), fast reading speed (100 tags per second), anti-interference properties, and lengthy service life (10 ± years). As a new management method, introduced in the automatic acquisition of characteristics, status, number of objects, it has been widely applied in logistics management, identity recognition, and access control. As shown in Figure 1, a typical UHF passive RFID system is comprised of an information service center, reader, and tag.

The RFID tag is made up of coupling components, chips, and antenna; each RFID tag has a unique electronic code, and the radio frequency signal of a particular frequency is sent by the RFID reader to the tag. When the tag is in the effective working area, induced current is produced in the tag due to coil coupling, and as a result, the tag is able to acquire energy to send its own coded information through a built-in radio frequency antenna. The information is then received by the receiving antenna and decoded by the signal processing module in the reader, which finally identifies the identity of the corresponding tag according to specific logic operation factors and sends the result to the information service center, which displays it on the software interface.

### 2.2. UHF Passive RFID Tag Design

**Figure 2:**
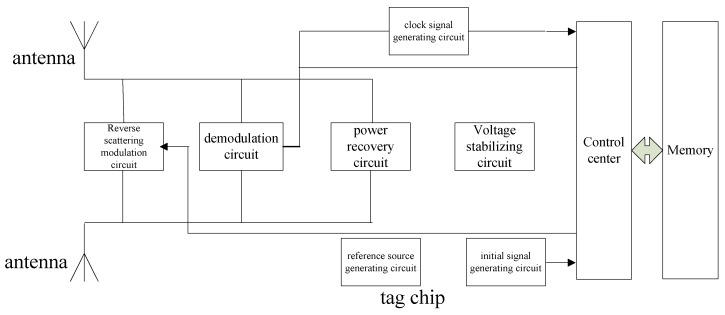
Schematic diagram of UHF passive RFID tag.

**Figure 3:**
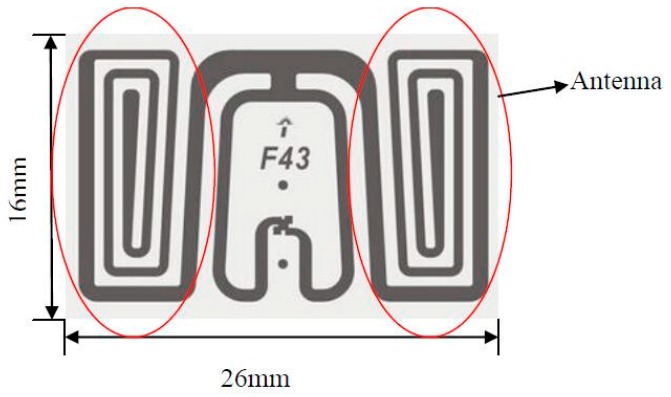
RFID tag Diagram.

The RFID tag in the system depicted above has the advantages of long reading distance (4 m) and high reading rate (response time is of 2.5 ms) at an ultra high frequency of 850–950 MHz. It also needs no external power supply, allowing for low cost and low power consumption. As shown in Figure 2, a passive RFID tag is comprised of several parts: the power recovery circuit, voltage stabilizing circuit, backscatter modulation circuit, demodulation circuit, clock signal generating circuit, initial signal generating circuit, reference source generating circuit, control unit and memory. Figure 3 shows a physical map of the tag, only 16 mm × 26 mm, and less than 1 mm in thickness, and its antenna. The radius of the large gear is 36 mm, which is sufficient to fix the tag completely on the gear.

The energy required for the passive RFID tag is fully derived from the energy of electromagnetic waves generated by the reader. The power recovery circuit therefore must convert the ultra high frequency signal received by the tag antenna to DC voltage.

The electromagnetic environment of the RFID tag is very complex, so a reliable voltage stabilizing circuit is required in order to ensure that the tag works properly in different electric fields. The modulation and demodulation circuits are the key components of the communication between the tag and reader.

The control unit of the RFID tag is a digital circuit that processes instructions. To make sure the digital circuit can be reset correctly to respond to the reader's instruction as soon as the tag is within the reader's reading distance, a reliable initial signal generating circuit is needed to provide the necessary reset signal for the digital unit.

### 2.3. Ultra High Frequency Passive RFID Reader Design

**Figure 4:**
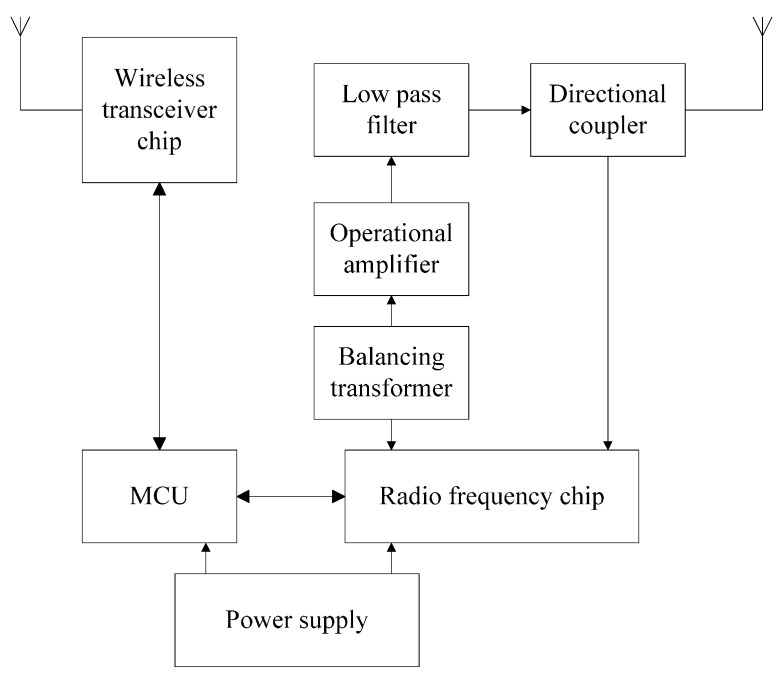
Schematic diagram of RFID reader.

As shown in Figure 4, the UHF RFID reader mainly consists of a wireless transceiver module, radio frequency module, and power supply. The wireless transceiver module consists of an MCU and wireless transceiver chip, which is responsible for communication between the radio frequency (RF) chip and MCU chip, controlling the RF chip to realize communication between the reader and tag and tag transmitting data through the antenna. The RF module is made of the RF chip, balancing transformer, operational amplifier, low pass filter, and directional coupler, which is used to process RF signals and transmit data to complete RFID tag reading data operation.

### 2.4. Bolt Loosening Monitoring System Based on RFID

**Figure 5:**
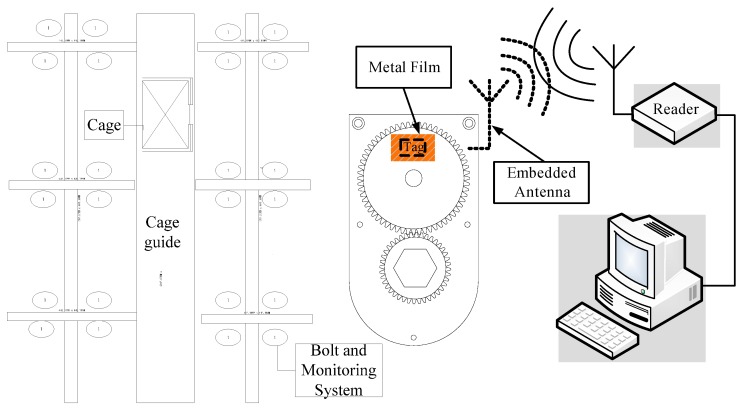
Measurement system principle diagram.

As shown in Figure 5, the monitoring system is fixed with the guide structure bolt, including two gears, a retaining plate, a tinfoil film, and an RFID tag. The reader and information service center are installed in the cage. While the cage is running to transport goods and staff into and out of the mine, the reader scans the cage guide bolts in turn automatically. Once bolt loosening occurs, the position and number of the loose bolts are displayed on the software interface, so that staff in the cage can easily find the loose bolts and quickly repair or replace them.

**Figure 6:**
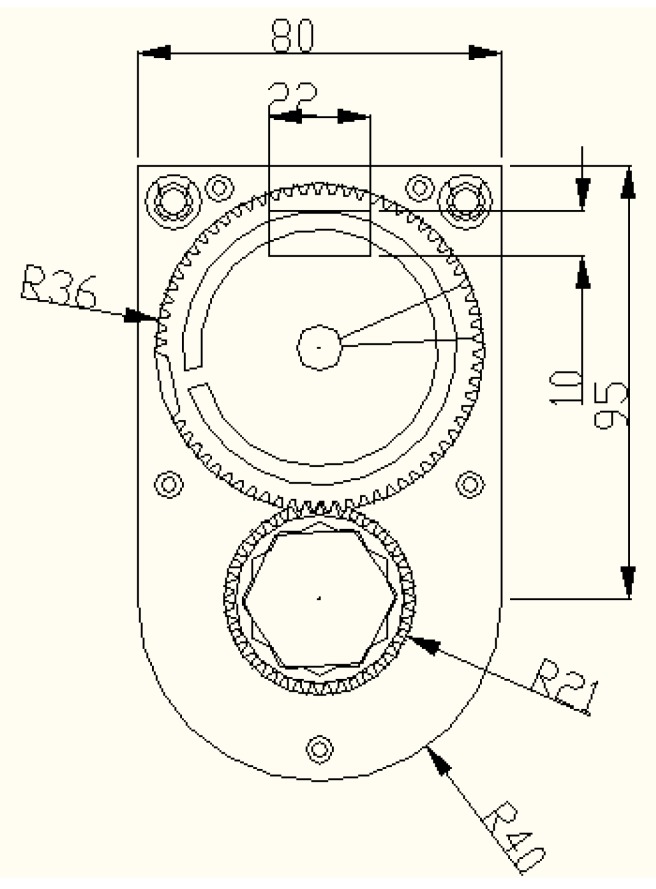
Structural diagram of gear system.

The gears and retaining plate in the monitoring system are made of POM plastic cement. The gear bonded to the tag needs to be sufficiently large to allow the tag fixed on it completely, while the monitoring system should be appropriately designed to be installed on the guide structure and bolt. We designed the monitoring system structure with these factors in mind as shown in Figure 6 and the unit in the diagram is mm. Metal film size was set to 20 mm × 30 mm with thickness less than 1 mm.

**Figure 7:**
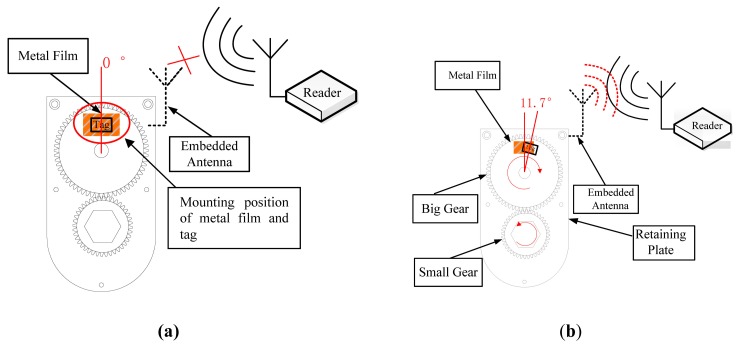
(**a**) Tag covered completely by metal film; (**b**) Rotational angle between tag and metal film exceeds 45°.

“Bolt loosening” in a bolt-nut system indicates where the rotation of the bolt and/or nut causes change in bolt tension and nut rotation angle [14]. It is relatively easy to measure the rotation angle of the nut, so in the proposed system, bolt loosening is judged accordingly. As shown in Figure 7a, the metal film was fixed on the retaining plate and the RFID tag bonded to the large gear. The mounting positions of the film and tag are shown in Figure 7a, where the angle of the film was designed to contact the corresponding position of the large gear and the tag was installed exactly in the middle of the film. The nut of the bolt to be detected (M18 product type) was fixed in the small gear’s center. The reader cannot receive radio-frequency signal if the tag is obscured by the tinfoil completely, because the tinfoil can absorb signals (which are electromagnetic waves). When the nut is loose, the small gear turns at the same angle as the nut fixed on the small gear, then under the gear transmission principle, the large gear rotates similarly to the small gear, rotating the tag on the large gear, however, since the film is attached to the retaining plate, which exposes its antenna. The radio-frequency signal including the corresponding nut’s information is then transmitted by the RFID tag to the RFID reader and the loose bolt’s location identified.

Experiments show that communication between reader and tag is successful when the nut rotational angle exceeds 20°, as shown in Figure 7b. The rotational angle between the tag and metal film can be calculated by the following formula:

(1) $$2\pi R\frac{x}{360^{\circ }}=2\pi r\frac{20^{\circ }}{360^{\circ }}$$

where *R* is the radiuses of the large gear; *r* is the radiuses of the small gear; and *x* is the rotational angle between the tag and metal film. The rotational angle between the tag and metal film is 11.7°, as shown in Figure 7b.

The bolt fastening force standard is based on the bolt design axial force. In the field, a 10% safety margin is added to the referential axial force. We set the allowable bolt rotation angle limit to an angle equivalent to the 10% design bolt axial force loss, and set the angle limit of the M18 bolt to 8° of the rotational angle. In practice, however, the angle limit may vary greatly according to the bolt material and base metal [1] In addition, due to the bolt joint loosening mechanism, minor looseness in the initial phase has a high probability of growing larger. Therefore, when rotational angle exceeds 20°, the proposed system can easily and immediately judge whether or not the bolt is loose.

## 3. RFID Reading and Writing Distance Test

### 3.1. Determinant Factors of RFID Reading and Writing Distance

Communication between the tag and reader is influenced by two main aspects. In the forward link, the electromagnetic wave energy sent by the reader should be controlled to ensure that the tag works successfully and sends accurate feedback data to the reader. In the back link, the reader must be sensitive enough to decode even weak signals from the tag.

At present, the transmitted power of a typical UHF passive RFID reader is generally less than 33 dBm and the minimum starting power of the tag is about −15 dBm; the reader’s sensitivity toward reflective signals from the tag is about −75 dBm. Maximum forward link attenuation is therefore about 48 dB. When the reflection efficiency of the tag is 3 dB, back link attenuation is about 57 dB. Of course, the bearable maximum attenuation of the forward link is less than that of the backward link, so the main limiting factor of the UHF passive RFID system is the forward link basically, the reader is able to receive reflective signals successfully as long as the tag works properly, and the reading distance of UHF passive RFID system can be calculated from the forward link.

Tag signal strength can be calculated as follows:

(2) $$P_{tag}=P_{t}G_{t}L_{path}$$

where *P_t_* is the transmitted power of the RFID reader; *G_t_* is antenna gain; *L_path_* is link loss when data is transmitted from the reader antenna to the tag, and *P_tag_* is the received maximum energy from the reader at an ideal point around the tag antenna (0 dB). *L_path_* can be derived as follows:

(3) $$L_{path}={\left(\frac{\lambda }{4\pi d}\right)}^{2}$$

Energy provided by the tag antennas must be more than the minimum operating power of tag. See the following:

(4) $$P_{c}=P_{t}G_{t}{\left(\frac{\lambda }{4\pi d}\right)}^{2}G_{tag}\tau \geq P_{cth}$$

where *P_c_* is energy obtained from the tag antenna by the tag; *G_tag_* is tag antenna gain; λ is wavelength; *d* is the distance between the reader antenna and tag antenna; τ is the power transmission coefficient determined by matching the tag antenna and tag chip, and *P_cth_* is the minimum power of the tag chip.

For a given tag with given frequency, the matching status and gain of the tag are certain. Therefore, the critical electromagnetic field intensity is determined by *P_cth_*, at which the tag is able to work properly:

(5) $$P_{tag,th}=\frac{P_{cth}}{G_{tag}\tau }$$

which is equal to the power received by the antenna at an ideal point.

Equation (6) is a combination of Equations (2), (4) and (5):

(6) $$P_{tag}=P_{t}G_{t}L_{path}\geq \;P_{tag,th}=\frac{P_{cth}}{G_{tag}\tau }$$

and *P_t_G_t_* can be replaced with equivalent omnidirectional radiation power (*EIRP*):

(7) $$P_{tag}=EIRP*L_{path}\geq P_{tag,th}$$

The read-write distance *R* can be calculated by Formulas (3) and (7):

(8) $$R=\frac{\lambda }{\mathrm{4\pi}}\sqrt{\frac{EIRP}{P_{tag,th}}}$$

where *EIRP* is the equivalent radiation power of the reader antenna, λ is the wavelength, *P_tag,th_* is the threshold that tag can respond normally, and *R* is reading distance. This formula demonstrates that the read-write distance of the RFID tag is indeed determined by the *EIRP* of the reader and the *P_tag,th_* of the tag.

### 3.2. RF Signal Strength Test

**Figure 8:**
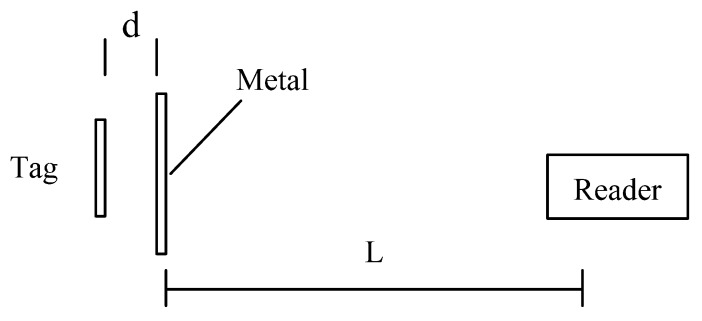
Schematic diagram of test device.

The performance of the RFID tag antenna is influenced by environmental factors [15,16]. The metal’s influence on tag performance and signal strength are important features of the proposed system. In theory, the reading distance is related to radio frequency of the system the higher the frequency, the greater the read-write distance. For example, the communication distance of a passive UHF RFID tag with radio frequency of 900 MHz can reach 10 m while a passive RFID tag with radio frequency of 16 MHz reaches only 10 cm at maximum. As depicted in Figure 8, we used a test device to measure the read range and read rate of the proposed system. This type of device has proven able to accurately reflect the influence of reading distance and tag read rate within differing surrounding environments in a coal mine [17,18,19].

**Figure 9:**
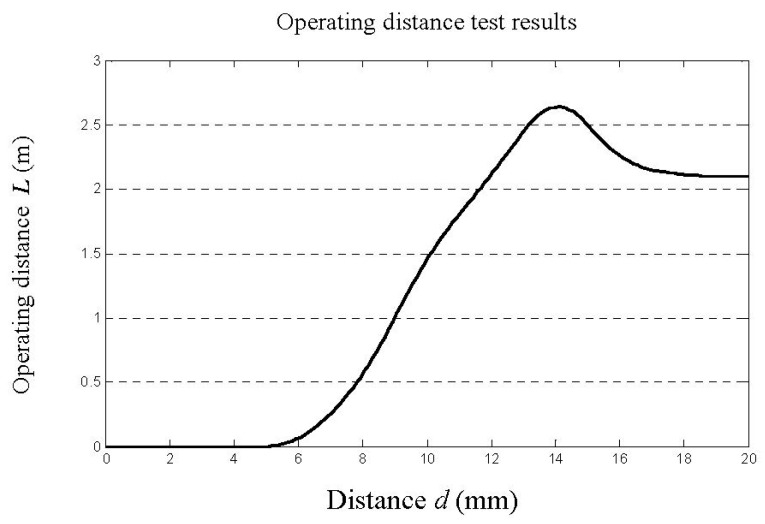
Diagram of test results.

The distance between the metal film and tag is *d*, and *L* is operating distance between the tag and reader, which changes as d changes. During testing, distance *d* was set to 0, 2, 4, 6, 8, 10, 12, 14, 16, 18 or 20 mm. Results are shown in Figure 9. We found that the tag was not identifiable when it was close to the metal, but as *d* increased beyond 8 mm, operating distance quickly increased until decreasing after *d* increased above 14 mm.

**Figure 10:**
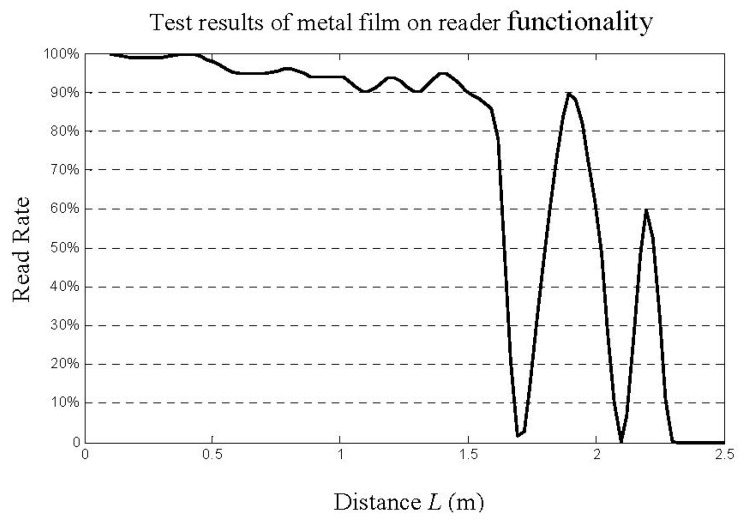
Results of signal strength test.

When the tag is attached to the metal or is very close to the metal surface, there is practically no RF field distribution in the space, and therefore no magnetic line to be cut by the tag antenna to measure the electromagnetic field energy, which prevents the passive RFID tag from functioning properly. We conducted an experiment accordingly to investigate the influence of metal on the reader by placing the test device on a metal plate in front of the reader antenna at varying distance *L*. The read rate shown in Figure 10 is the success rate at which the reader received radio-frequency signals from the tag as *L* changed.

## 4. System Software Design

**Figure 11:**
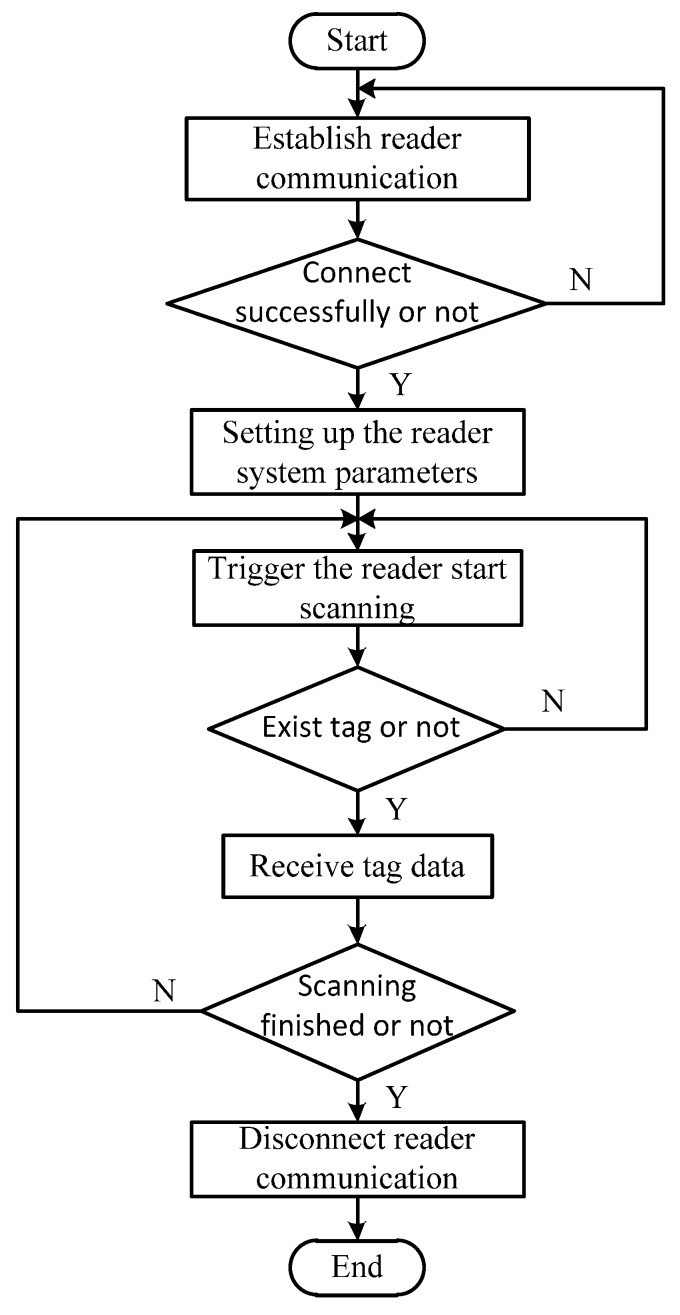
Program flow chart of interface software system.

**Figure 12:**
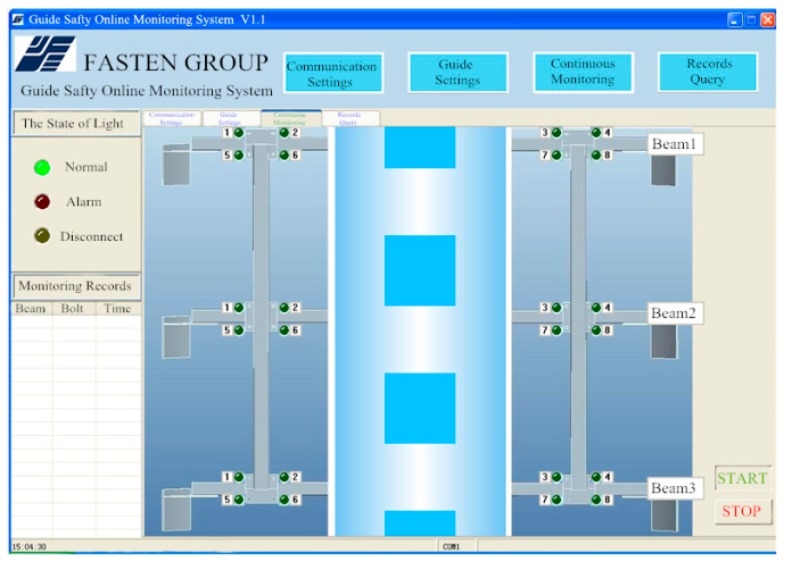
Continuous monitoring software.

A program flow chart of the interface software system is shown in Figure 11. The program effectively controls the RFID reader to scan tags in the range of communication (about 2.5 m) to detect bolt loosening. The proposed software is capable of monitoring thousands of bolts simultaneously, where an alarm is immediately triggered if the state of any bolts becomes abnormal. The alarm can be a siren, flashing light, or other signal. As shown in Figure 12, by clicking the START button on the software interface, the reader begins to scan the tags. The tag sends information to the reader when a corresponding bolt loosens, then its corresponding symbol on the screen turns red to sound a warning. When the 24 bolt symbols displayed on the screen are green, all 24 bolts are in normal state; the location and number of loose bolts are indicated clearly and immediately by the red lights on the monitoring software interface.

All the information retrieved from the tags and commands sent to the tags by the reader are recorded in the computer database by the software automatically. The inventory and history files from the tag memory are also read routinely, so data integrity can be guaranteed by comparing new signals against the database records. The database is made compatible to existing systems at each storage site, and the data can be accessed by privileged remote users through a secured network. The search function is extremely useful for inventory management, especially when the size of the inventory is large.

## 5. Confirmatory Experiments and Comparative Study

### 5.1. Confirmatory Experiment

**Figure 13:**
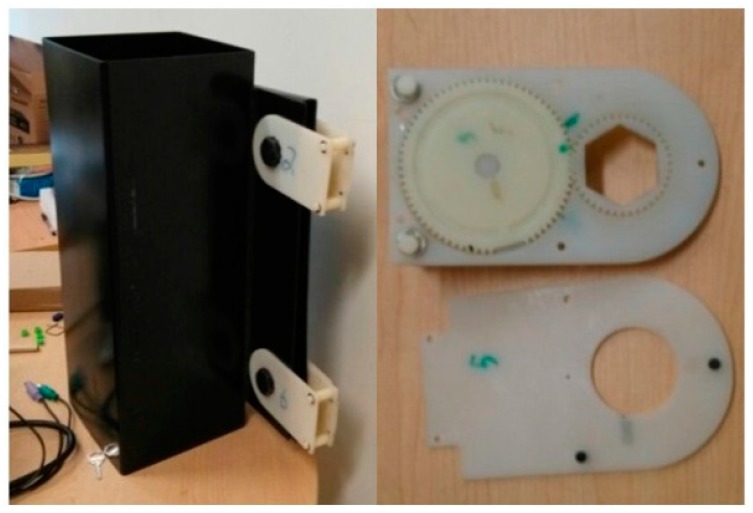
Monitoring test of guide simulation structure.

The guide simulation structure we used to verify the proposed system’s effectiveness is shown in Figure 13. The guide and plate are secured with M18 bolts, labeled 1, 2, 5, 6, respectively. The height of the guide simulation device is 50 cm, and it is 10 cm in both length and width.

When the bolt is tightened, the tag is completely blocked by the metal film and tag information cannot be read by the reader. When the nut of the bolt is loose, the tag turns with the nut, exposing its antenna and sending information read by the reader to the monitor. The position of the loosened bolt is then displayed on the monitoring device in the monitor room.

During the test, Bolt 2 sounded an alarm on the monitoring device once the rotational angle of its nut exceeded 20°. When the rotational angles of Nut 2 and Nut 6 were more than 20°, Bolt 2 and Bolt 6 sounded alarms on the monitoring device. In other words, the threshold for bolt loosening in the monitoring software is a rotational angle of 20°, at which point a sound and light alarm alerts the user. These experimental results demonstrate the feasibility and effectiveness of the proposed system.

### 5.2. Comparative Experiment

**Figure 14:**
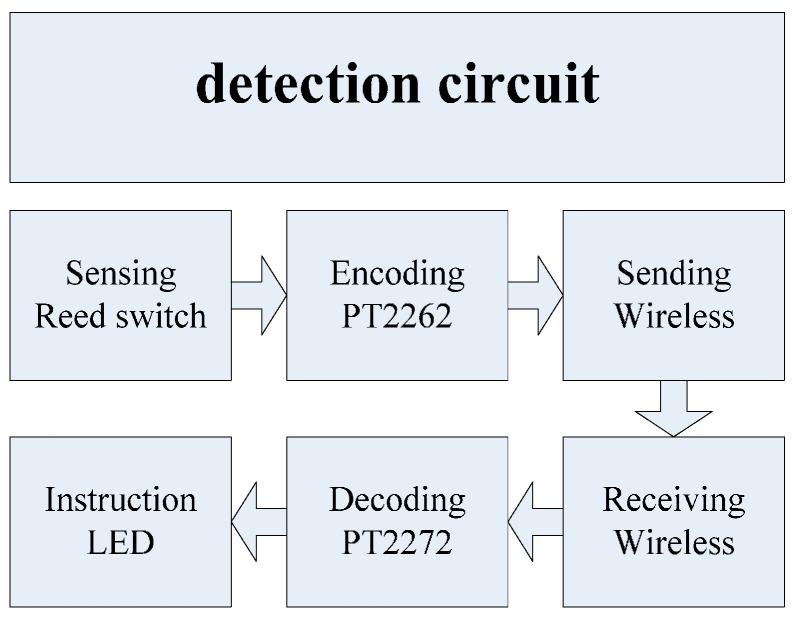
Schematic diagram of reed switch bolt loosening detection system configuration [20].

For the sake of comparison, we tested the proposed system against another effective system based on reed switching developed to monitor multipoint bolt loosening is presented in this paper [1,20]. The detection circuit is comprised of a reed switch, wireless receiver and transmitter circuits with 315 MHz operation frequency, encoder PT2262, decoder PT2272, and instruction LED, as shown in Figure 14.

**Figure 15:**
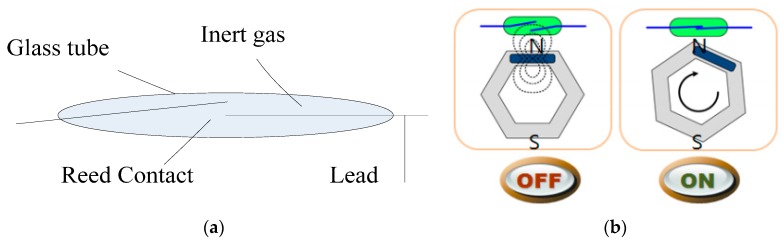
(**a**) Monitoring reed switch [20]; (**b**) Monitoring principle of reed switch system [20].

In the comparable monitoring system, the reed switch which consisted of two thin metal contactors encapsulated in a glass tube, serve as the sensor for bolt loosening. As shown in Figure 15a, a special magnetic sensing switch turns on or off according to changes in the intensity of the surrounding magnetic field. Once the reed switch is turned on, the wireless transmitter circuit begins to work and the corresponding LED illuminates. The operating principle of reed switch is depicted in Figure 15b, where when the magnetic field remains in equilibrium, the switch is off, and when nut of the bolt rotates, the field is biased, and the switch flips on. As soon as the reed switch is closed, the test node sends the message that the bolt has loosened to the receiving terminal.

**Figure 16:**
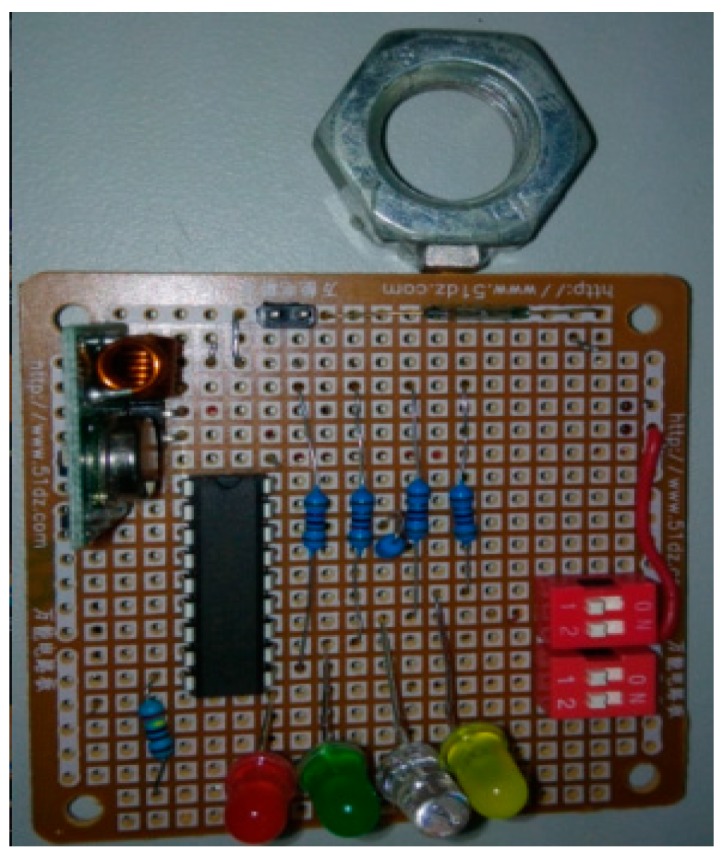
Bolt loosening detection sensor circuit system [20].

In the comparative experiment, a circuit board with four nuts was designed as an experimental model, each nut was assigned a number, and a square magnet was attached to one side of the screw nut so that when the screw nut was rotated, the magnet on the bolt rotated accordingly, changing the magnetic field and triggering the reed switch detection system to sound an LED alarm. The experiments showed that the reed switch alarm sounded when nut rotation angle exceeded 10°–12° (Figure 16).

### 5.3. Comparative Reaserch Conclusions

We compared the two systems as listed in Table 1, where System A is the proposed system and System B is the reed switch system. During the experiment, single cost was 100 test nodes plus the signal receiving system—In other words, one RFID reader in A and two receiving terminals in B. *S* indicates the area of test node plane and *P* the power dissipation of the working test node. Success rate was defined as the reliability of the system, and monitoring angle is the nut rotational angle able to be detected by the systems.

**Table 1:** Results of two systems.

System	Single Cost/RMB	Cost of 100 Nodes/RMB	P/mW	S/cm^2^	Success Rate	Monitoring Angle
A	0.3	830	0	1	99%	Approx. 20°
B	10	1020	300	16	95%	10°–12°

We found several points in common between the two systems—Most importantly, that both are quite reliable, because they both judge bolt loosening based on change in the state of the bolts rather than signal extraction and analysis. The two systems also are both able to monitor hundreds of bolts simultaneously in the working environment.

The RFID tag system did show a few notable advantages compared to the reed switch system. First, less power is needed to run it because there is no battery in the testing node. The proposed system is also cheaper (Table 1), and the test node of the reed switch system needs more complex circuitry to process signals while only one tag is needed in the node of the proposed system. The measurement accuracy of System B is superior to that of System A, which can detect smaller rotational angle—We found that the proposed system judges bolt loosening effectively regardless of the relatively larger nut angle, however.

In a typical coal mine cage guide, the proposed method represents an effective and practical approach to loose bolt monitoring. The proposed system also, importantly, can be readily optimized by improving its measurement accuracy, it is able to detect smaller rotational angles by increasing the radius ratio of the small gear and large gear in the system. Due to constraint related to the guide structure, however, the designed system can only detect bolt loosening in this specific application when the nut rotational angle exceeds 20°.

## 6. Conclusions

The bolt monitoring system proposed in this paper and the experiments conducted to investigate its effectiveness can be summarized as follows:

(1) An innovative approach to the loose bolt problem in coal mine cage guide structure, which is caused by harsh environment and many other factors, was developed in this study. The proposed monitoring system consists of a bolt gearing system, passive UHF RFID tags, metal film, reader, and monitoring software, the system utilizes passive RFID technology, as discussed at length above. Within sufficient range of communication, the reader sends radio-frequency signals to the RFID tag, which is powered by the electromagnetic wave due to coil coupling principle before sending information back to the monitor.
(2) To construct the system, an RFID tag was fixed on a large gear and a tinfoil metal film was fixed on the retaining plate. When the RFID tag is covered by the tinfoil, it is unable to communicate with the reader, the nut to be detected is fixed in the small gear’s center, so once the nut loosens, the rotation of the small gear rotates the large gear, exposing the tag antenna which then sends a radio-frequency signal from the corresponding bolt to the RFID reader and displayed on the monitoring software interface.
(3) We examined the reading distance between the reader and tag and effective distance between the tag and metal film, as well as the relationship of reading rate and reader and metal film distance all in terms of the proposed system’s feasibility and potential to be optimized successfully. A similar, proven-effective multipoint bolt loosening monitoring system based on reed switch was also tested for the sake of comparison to evaluate strengths and weaknesses of the proposed system.
(4) Constrained by installation form of coal mine guide structure, the proposed system can only detect bolt loosening when nut rotational angle exceeds 20° though, the system can theoretically be further optimized by changing the radius ratio of the two gears. To this effect, the proposed method represents a good reference for use in other applications that employ bolt-nut systems.
